# Risk Factors of Anesthesia-Related Mortality and Morbidity in One Equine Hospital: A Retrospective Study on 1,161 Cases Undergoing Elective or Emergency Surgeries

**DOI:** 10.3389/fvets.2019.00514

**Published:** 2020-01-22

**Authors:** Chiara Laurenza, Lèa Ansart, Karine Portier

**Affiliations:** ^1^Section of Anesthésiologie, Université de Lyon, VetAgro Sup, Marcy l'Étoile, France; ^2^GREAT, Laboratoire Carmen, INSERM U1060, INRA U1235, INSA Lyon, Université Claude Bernard Lyon 1, Villeurbanne, France

**Keywords:** horse, equine, anesthesia, mortality, perioperative complications, risk factors

## Abstract

A retrospective analysis was performed to determine mortality and morbidity rates for elective and emergency cases in an equine university teaching hospital. It investigated the effect of horse-, anesthetic-, timing, and clinician experience-related variables on anesthetic complications. In total, 1,161 horses undergoing general anesthesia between January 2012 and December 2016 were included in the study. Patient information and details of the anesthetic, recovery period and immediate complications were retrieved from an archival database. Statistical analysis of qualitative and quantitative factors affecting anesthetic complications was performed using an univariable and multivariable ordinal logistic regression. Odds ratio of variables primarily affecting mortality and complications were calculated. Statistical significance was set at *p* < 0.05. General anesthesia-related global mortality rate was 1.4% (95% CI [7.1–10.4]) but was only 0.96% (95% CI [0.44–1.82]) for non-colic cases. The complication rate was 17.5% (*n* = 204; 95% CI [15.2–20.0]) of which 46.9% [39.4–54.5] were neuromuscular, 22.6% [16.7–29.5] were respiratory, 15.8% [10.8–22.0] were systemic, 13.6% [8.9–19.5] were cardiovascular, 1.1% [0.1–4.0] were other complications. Ninety two percent of complications occurred during recovery. Major risk factors for mortality and complications included high weight, surgeon experience, increasing age, high ASA score, long duration of anesthesia, quality of induction, lateral recumbency, orthopedic surgery, and hypotension. In these models, colic surgery did not influence the rate of any complications.

## Introduction

Equine anesthesia has become a routine practice in most horse hospitals. However, the anesthetic risk of mortality does not seem to have decreased over time (1). The most severe complications being fatal. In 2002, the Confidential Enquiry into Perioperative Equine Fatalities (CEPEF), a prospective observational epidemiological multicenter study, found a risk of death (up to 7 days after anesthesia) to be 0.9% for healthy horses (2). The most recent study, performed in a single equine university teaching hospital between 2010 and 2013, published an identical mortality rate (0.9%) for elective cases (up to the return of the horse to its stable) (3). Since the mortality rate is more easily calculated, it is more often studied than the morbidity rate. Nevertheless the fact remains that the published results are extremely variable from one study to another (4).

While caution in comparing the results of such studies should remain the rule (due to differences, for example, in study design, hospitals, sample size, selection of cases, observation time, definition of the outcomes), the issue of decreasing anesthetic mortality over time has always been debated and compared across species (1). Mortality rate in healthy horses is higher than in healthy dogs (0.05%), cats (0.11%), and rabbits (0.73%) (5). The death rate in humans is more than a thousand times smaller (0.69 10^−3^%) than in animals. In France, an investigation into millions of procedures showed that mortality was divided by 10 in 15 years between 1986 and 2000 (6). Since the mortality and morbidity rate is high in equine anesthesia compared with human anesthesia, it should be possible to make a risk analysis using smaller samples than in humans (1). The reasons for these differences in rates and trends between species are multiple. The heavy weight of the horse and its poor tolerance to depression of cardiovascular and respiratory functions could explain a higher rate of complications in this species (4). The recovery phase is described as the riskiest phase of equine anesthesia. This may be related to the anatomy and behavior of the horse and to lack of monitoring and cardiovascular support in the recovery box (2).

However, the mortality and morbidity rates related to anesthesia should not be directly correlated with safety level. There is little information on morbidity, but even if the mortality rate has not changed a lot, some progresses have been made in terms of monitoring, anesthesia equipment, training and information of equine veterinarians (7, 8). In parallel surgical procedures have become more complex and are performed on patients at higher risk (9). Therefore, it is reasonable to assume that in the near future the decrease in anesthetic mortality and morbidity will be related to an improvement in safety.

One of the best ways to improve security is to inform equine anesthesiologists and surgeons in order to guide the logic of their interventions/professional practices made in the interest of anesthetic safety. The effectiveness of guidelines to reduce anesthetic risk is controversial (1). Nevertheless, attempts to reduce the risk of anesthesia are still possible by continuing to document mortality and morbidity rates and describing any factor associated with an increased rate of death/post-operative complication. The analysis of triggering causes, favoring or simply associated with these events, is essential. Risk factors (associated with increased rate of death) mostly reported in the equine literature are American Society of Anesthesiologists (ASA) physical status, age, the type of surgery, prolonged duration of anesthesia and out-of-hour surgery (4).

The primary aim of this retrospective single-center study was to document the mortality and morbidity rate associated with elective and emergency procedures in horses undergoing general anesthesia in our clinic. The secondary aim was to investigate the intrinsic and extrinsic factors that determined anesthetic mortality and morbidity from the pre-operative clinical examination up to the end of recovery. The complications that occurred between the end of recovery and the 48 h postoperative were also described.

## Materials and Methods

The medical records of all horses (*n* = 1,161) that underwent general anesthesia at the Equine Teaching Hospital of Lyon for elective and emergency surgeries between January 2012 and December 2016 were collected. Patient information and details of the anesthetic management, recovery period and immediate complications within 48 h postoperatively were obtained from the anesthesia report, the surgical report and the complication report if applicable.

The information (and their categorization) recorded from these documents were as follows: details about the horse: breed (pony, sport horse, draft horse, racehorse, other), age (year), sex (male, gelding, mare), body mass (kg), pregnancy (yes/no), ASA physical status classification (1–5). The types of surgery were arthroscopy, colic, castration, skin tumor resection, orthopedic surgery (other than arthroscopy), head surgery, septic surgery, others. The distribution of surgeries in these categories is detailed in Table 1. The experience of the surgeon (resident vs. senior), whether anesthesia was done for elective or emergency procedure, fasting (yes/no), time of the year (first or second semester), time of the week (weekend or not), time of the day (8 a.m.−8 p.m./8 p.m.−00 a.m./00 a.m.−8 a.m.) were also noted.

**Table 1:** Distribution of the 1,161 surgeries, performed at the Equine Teaching Hospital of Lyon between January 2012 and December 2016, in each of the eight categories defined in the study.

**Surgery categories**	**Surgical procedures**	**Number of Cases**
Arthroscopy	Bursoscopy, tenoscopy, intra-articular lavage	253
Colic	Colic, umbilical hernia, inguinal hernia, dystocia, cesarean section	229
Castration	Castration	199
Skin tumors resection	Sarcoids resection	135
Orthopedic surgery	Fracture, arthrodesis, angular deviation, cast change, neurectomy, osteosynthesis material removal, keratoplasty, sequestrectomy, cyst excision, periosteal elevation, surgical correction of patellar luxation	108
Head	Transarterial coil embolization, sinusotomy, ethmoid hematomas, dentistry, ophthalmic surgery, laryngeal surgery	99
Septic surgery	Wounds, foreign body and abscess debridement, umbilical infection, patent urachus	98
Others	X-Ray, computer tomography, myelography, cerebrospinal fluid puncture, cisplatin treatment, stem cells injection	40

The conventionally used anesthesia protocol (administered to the majority of healthy horses) included tranquilization with acepromazine, premedication with an alpha 2 agonist (romifidine, detomidine, or xylazine) and an opioid (morphine or butorphanol), induction with diazepam and ketamine and maintenance with isoflurane or sevoflurane in 100% O_2_. Inspiratory positive pressure ventilation (IPPV) was performed. The volume was adjusted to maintain normocapnia with a maximal positive inspiratory pressure at 35 cmH_2_O. Occasionally, anesthesia was maintained by an infusion of guaiphenesin with ketamine and xylazine for short-term procedures. Dobutamine was used to maintain mean arterial blood pressure (MABP) above 70 mmHg, if necessary. Analgesia was provided with a non-steroidal anti-inflammatory drug (phenylbutazone or flunixin), an opioid (as described above) and local anesthetic (lidocaine) when possible. The analgesic protocol was sometimes complemented by constant rate infusion (CRI) of morphine and/or ketamine and/or lidocaine and/or dex/medetomidine, at the discretion of the anesthetist. Horses presented in a state of physiological shock received a protocol of resuscitation and the anesthesia was adapted to the case (for example without acepromazine).

The following anesthesia data (and categorization) were recorded from the anesthesia sheet: experience of the anesthetist (senior vs. resident), body position (dorsal vs. lateral), duration of anesthesia (from induction to entrance in the recovery box), whether dobutamine was administered during the second half of the surgery (yes/no), premedication and induction protocol (conventional vs. other), administration of a CRI (lidocaine or alpha 2 agonist or none), anesthetics used for maintenance (isoflurane or sevoflurane or injectable). Calcium plasma concentration during colic surgery (mean concentration in mEq/L), pH (mean value), PaO_2_/FiO_2_ ratio (mean value), invasive MABP (mean value), hypotension index [HI = 1/12∑(70-MAP)] (10) were also recorded.

Recoveries were performed in a rubber-padded recovery box. Intranasal phenylephrine was administered before moving the horse to the recovery box. Oxygen was administered either by a demand valve (in case of apnea) through the orotracheal tube or by insufflation through a nasopharyngeal or orotracheal tube.

The characteristics of the recovery that were recorded were as follows: whether the recovery was assisted or not, the duration (from entrance in the recovery box to standing) and the quality of recovery (with a score using dichotomous objective descriptors (11).

Cases were also classified according to the outcome of the procedure: alive, dead, or euthanized. Total mortality was defined as all cases that were euthanized or died within the period from the induction of anesthesia to the return to the hospitalization box. The dead were also divided according to the primary cause of death. We defined anesthetic mortality as all deaths considered due solely to anesthesia that unexpectedly occurred during surgery, and euthanasia due to peri-anesthetic complications. Non-anesthetic mortality was then defined as all deaths due to a cause not attributable to anesthesia, and euthanasia related to inoperable lesions.

Non-fatal complications were defined as complications that did not induce death within the recovery period. They were recorded and classified according to 4 categories: respiratory, neuromuscular, cardiovascular and systemic. The overall non-fatal complication rate for the observation period was also calculated (number of non-fatal complications related to anesthesia divided by the total number of anesthesia).

### Statistical Analysis

Statistical analysis was performed using R (https://www.rstudio.com/) statistical software. Qualitative data were described using frequency distribution. Quantitative data were checked for normality of distribution using a Shapiro Wilk test. They were all normally distributed and expressed as the mean ± standard deviation (SD).

The relationships between possible risk factors and mortality or complications were tested using Pearson's chi-squared test or Fisher's test for qualitative variables and Student's *t*-test for quantitative variable. Significance level was set at *p* < 0.05 for univariate analysis.

Predictors which have been found significant at *p* < 0.2 by the univariate logistic regression were tested in a multivariable model. The Akaike information criterion (AIC) was used to estimate the relative quality of the models as a mean for model selection. The best model was also considered the one with the smallest possible number of variables. The links between explanatory variables were also studied in order to identify confounding factors, using a linear regression analysis, a correlation or a Chi-Square test.

## Results

The results presented here concern observation until the return to the hospitalization box immediately after recovery. The complications observed in the 48 h that followed are described in the discussion. We could not include this observation period in the statistical study because of the uncertainty of the recruitment, this period not being under the control of the anesthetists. In addition, some horses were discharged before 48 h post-surgery.

### Description of the Sample

A total of 1,161 horses underwent general anesthesia at the Equine Teaching Hospital of Lyon for elective and emergency surgeries between January 2012 and December 2016.

To study the risk factors for each type of anesthetic complication those who died for surgical reasons (*n* = 84) were excluded from this count. The medical records of 438 mares, 293 geldings, 346 stallions (total 1,077) were therefore studied (*n* = 236 in 2012, *n* = 214 in 2013, *n* = 224 in 2014, *n* = 214 in 2015, *n* = 188 in 2016). They had a body mass of (median, range) 481 [38–730] kg and were aged 5 [1–35] years. The distribution by breed category was as follows: saddle horses (*n* = 752; 70%), ponies (*n* = 132; 12%), racehorses (*n* = 104; 10%), horses of undetermined breed (*n* = 58; 5%), draft horses (*n* = 31; 3%). Horses ASA physical status was divided into 5 categories [ASA1 *n* = 118 (11%), ASA2 *n* = 631 (58%), ASA3 *n* = 152 (14%), ASA4 *n* = 104 (10%), ASA5 *n* = 72 (7%)].

Most of the surgeries were elective (*n* = 802; 74%) whereas 275 (26%) were emergency procedures. These surgeries were performed by a diplomate of the European College of Veterinary Surgery (ECVS) (*n* = 662; 61%) or a resident (*n* = 369; 34%), for 46 (5%) surgeries the qualification of the surgeon was not specified. Horses underwent arthroscopy (*n* = 252; 23%), castration (*n* = 189; 17%), colic surgery (*n* = 160; 15%), skin tumor resection (*n* = 136; 13%), surgery of the head (*n* = 108; 10%), orthopedic surgery (*n* = 104; 10%), septic surgery (*n* = 94; 9%), other type of surgery (*n* = 34; 3%).

The duration of anesthesia was (median, range) 135 [0–495] min. The duration of recoveries was (median, range) 45 [5–315] min. Recoveries were unassisted for 410 cases whereas they were assisted (head and tail ropes or Anderson sling for fractures) in 667 cases. Anesthesia was performed by a diplomate of the European College of Veterinary Anesthesia and Analgesia (ECVAA) in 343 (32%) cases and by a resident in 681 (63%) cases. The qualification of the anesthetist was not specified for 53 (5%) surgeries.

### Mortality Rates

Total mortality rate was 8.6% (*n* = 100/1161; 95% CI [7.1–10.4]). Non anesthetic mortality rate was estimated at 7.2% of the total case number (*n* = 84/1161; 95% CI [5.9–9.0]). Anesthetic mortality rate was 1.4% (*n* = 16/1161; 95% CI [0.7–2.1]). A total of 932 horses underwent surgeries other than emergency abdominal surgeries. The non-colic anesthetic mortality rate was 0.96% (*n* = 9/932; 95% CI [0.44–1.82]). The anesthetic mortality rate of horses presented for colic (*n* = 229) was 3% (7/229). The rate of euthanasia due to inoperable lesion of horses anesthetized for colic was 30% (68/229). The total mortality rate for horses presented for colic was therefore 33% (75/229).

The details of the causes of mortality are presented in Table 2.

**Table 2:** Details of primary causes of anesthetic and non-anesthetic mortality of horses which were presented at the Equine Teaching Hospital of Lyon between January 2012 and December 2016 for colic and non-colic surgery.

**Number of deaths**	**Euthanasia/Spontaneous Death**	**Cause of death**	**Death attributed to**	**Reason of anesthesia**
68	Euthanized	Inoperable lesions	surgery	Colic surgery
3	Euthanized	Inoperable lesions	surgery	Diagnostic Imaging
2	Euthanized	Inoperable lesions	surgery	Wound debridement
2	Euthanized	Inoperable lesions	surgery	Skin tumors resection
2	Euthanized	Inoperable lesions	surgery	Fracture surgery
1	Euthanized	Inoperable lesions	surgery	Arthroscopy
1	Euthanized	Inoperable lesions	surgery	Septic arthritis
1	Euthanized	Inoperable lesions	surgery	Thoracotomy
1	Euthanized	Inoperable lesions	surgery	Articular lavage
1	Euthanized	Inoperable lesions	surgery	Dystocia
1	Euthanized	Inoperable lesions	surgery	Cisplatin treatment
1	Euthanized	Inoperable lesions	surgery	Cast placement
2	Euthanized	Failure of osteosynthesis material	anesthesia	Fracture surgery
1	Euthanized	Fracture at recovery	anesthesia	Colic surgery
1	Euthanized	Fracture at recovery	anesthesia	Fracture surgery
1	Euthanized	Severe myopathy	anesthesia	Colic surgery
1	Euthanized	Myelomacia	anesthesia	Castration
1	Euthanized	Myositis+Vestibular syndrome	anesthesia	Transarterial coil embolization
1	Euthanized	Fall	anesthesia	Fracture surgery
1	Euthanized	Severe myopathy	anesthesia	Sinusotomy
1	Euthanized	Weakness syndrome+Myopathy	anesthesia	Colic surgery
1	Euthanized	Weakness syndrome+Paralysis	anesthesia	Arthroscopy
1	Dead	Cardiorespiratory arrest at induction	anesthesia	Colic surgery
1	Dead	Cardiorespiratory arrest at recovery	anesthesia	Colic surgery
1	Dead	Cardiorespiratory arrest at recovery	anesthesia	Osteosynthesis material removal
1	Dead	Intraoperative cardiorespiratory arrest	anesthesia	Colic surgery
1	Dead	Myopathy, prolonged recovery, respiratory arrest	anesthesia	Colic surgery

### Factors Associated With Increased Mortality

Among the studied factors described in the material and method section, increased age (*p* = 0.04), higher ASA physical status (*p* < 0.01), colic surgeries (*p* < 0.01), orthopedic surgeries (*p* = 0.01), seniority of the surgeon (*p* = 0.02), emergencies (*p* = 0.04), longer anesthetic duration (*p* < 0.001), worst quality of recovery (*p* < 0.001) were significantly associated (univariable logistic regression) with increased mortality. Other factors had no significant effect on survival.

Predictors that were significant at *p* < 0.2 were tested in a multivariable model. The best model for explaining anesthetic risk included 5 variables. The risk of anesthetic mortality was multiplied by 1.5 when the weight of the horse increased by 100 kg; 2.1 when the ASA score increased by one point; by 5.5 when the surgeon who operated was a senior and by 9.8 during orthopedic surgery. The risk was decreased by 0.4 when the anesthesiologist used dobutamine during the second half of the surgery. Odd-ratio of the explanatory variables for the risk of anesthetic mortality are presented in Table 3.

**Table 3:** Results of the multivariable model showing odds-ratio of the explanatory variables for the risk of anesthetic mortality, neuromuscular, respiratory, systemic and cardiovascular complications.

**Variable**	**Anesthetic mortality**	**Neuro muscular complication**	**Respiratory complication**	**Systemic complication**	**Cardiovascular complication**
Senior surgeon	5.49 [1.05–10.10]	2.14 [1.10–4.44]	2.58 [1.04–7.79]		
Weight/100	1.54 [1.02–2.60]	1.26 [1.00–1.63]	1.57 [1.08–2.42]		
Age			1.71 [0.91–3.06]		1.08 [1.00–1.15]
Anesthesia duration		1.35 [1.05–1.71]	1.49 [1.12–1.94]		
ASA	2.10 [1.27–3.56]			1.93 [1.42–2.61]	
PaO_2_/FiO_2_			0.66 [0.47–0.92]		0.52 [0.33–0.88]
Dobutamine	0.37 [0.11–1.12]				
Orthopedic surgery	9.82 [2.55–38.04]				
Arthroscopy		0.55 [0.26–1.06]			
IBP/10		1.25 [0.95–1.63]			
Induction quality		0.82 [0.73–0.93]			
Lateral recumbency					4.89 [1.78–13.45]

### Non-fatal Complication Rates

**Figure 1:**
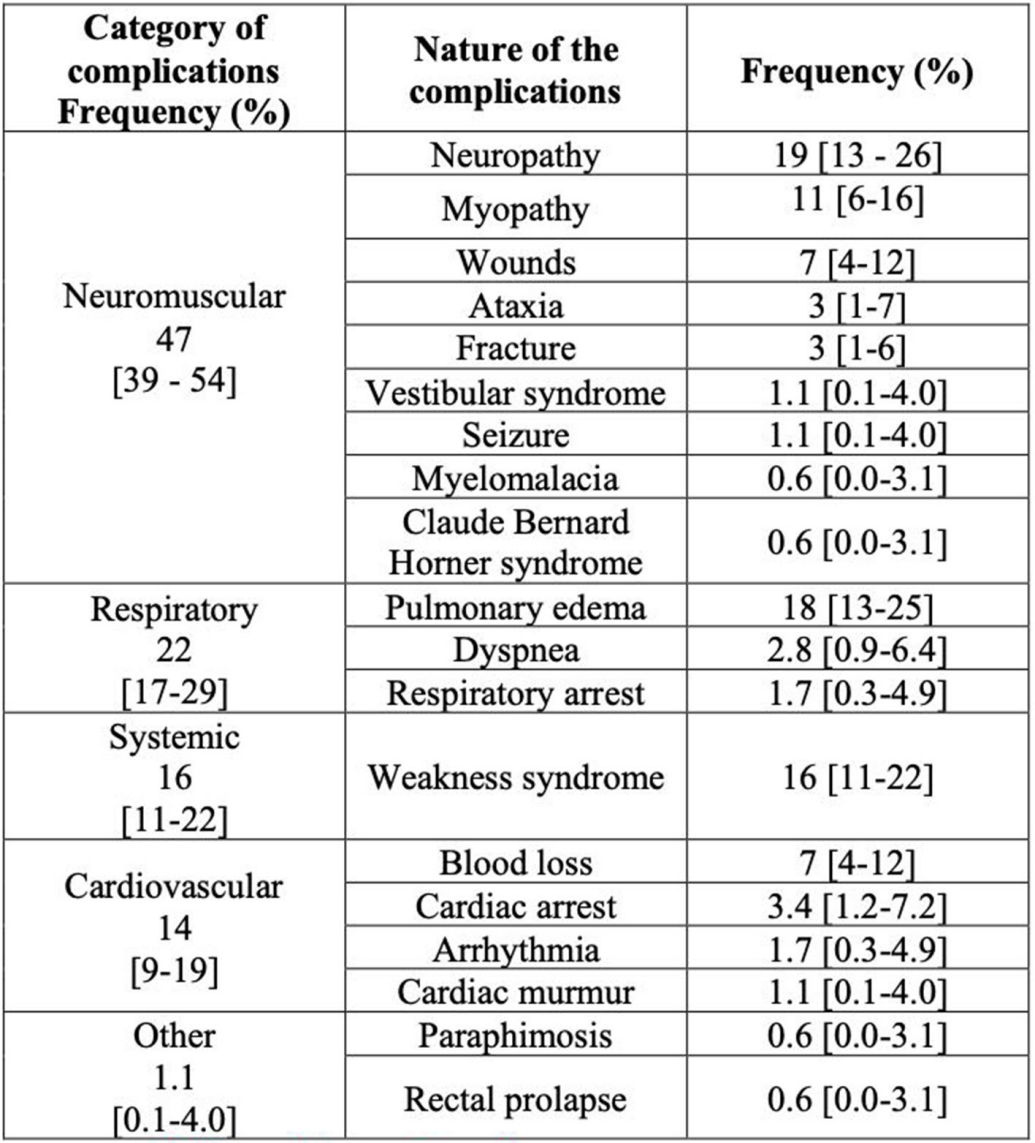
Details and frequencies of the anesthetic complications suffered by horses anesthetized between January 2012 and December 2016 at the Equine Teaching Hospital of Lyon.

The complication rate was 17.5% (*n* = 204; 95% CI [15.2–20.0]) of which 46.9% [39.4–54.5] were neuromuscular, 22.6% [16.7–29.5] were respiratory, 15.8% [10.8–22.0] were systemic, 13.6% [8.9–19.5] were cardiovascular, 1.1% [0.1–4.0] were other complications. The details of the nature of the complications and their frequencies are presented in Figure 1. Almost all the complications (92 [87–96] %) occurred during recovery.

Risk factors were associated with different types of complication (univariable logistic regression).

Neuromuscular complications were significantly associated to increased age (*p* < 0.01), increased weight (*p* < 0.01), increased ASA physical status (*p* = 0.01), colic surgery (*p* = 0.02), orthopedic surgery (*p* < 0.01), surgeon experience (*p* < 0.001); emergency vs. elective procedure (*p* = 0.03), when horses were not fasted (*p* = 0.04), lateral recumbency (*p* = 0.04), longer duration of anesthesia (*p* < 0.001), lower plasma calcium concentration (*p* < 0.001), higher hypotension index (*p* < 0.01), worst quality of recovery (*p* < 0.00001), longer duration of recovery (*p* < 0.00001).

Respiratory complications were significantly associated to the year (*p* < 0.01), increased age (*p* < 0.001), higher weight (*p* < 0.001), higher ASA score (*p* < 0.001), colic surgery (*p* < 0.00001), surgeries other than castration (*p* = 0.03), surgeries other than skin tumor resection (*p* = 0.04), surgeon experience (*p* < 0.01), emergency procedure (*p* < 0.01), end of the day and night (*p* < 0.00001), longer duration of anesthesia (*p* < 0.0001), lower Pa0_2_/FiO_2_ ratio (*p* < 0.001), worst quality of recovery (*p* < 0.01), and longer duration of recovery (*p* < 0.01).

Systemic complications were significantly associated to increased ASA score (*p* < 0.0001), colic surgery (*p* < 0.001), emergencies (*p* < 0.01), longer duration of anesthesia (*p* = 0.01), worst quality and longer duration of recovery (*p* < 0.00001).

Cardiovascular complications were significantly associated to increased age (*p* < 0.001), lateral recumbency (*p* = 0.03), lower PaO_2_/FiO_2_ ratio (*p* = 0.02).

### Variables Explaining the Risk of Non-fatal Complications in Multivariate Models

The risk of neuromuscular complications was multiplied by 1.3 when the weight of the horse increased by 100 kg, by 1.3 when the duration of anesthesia increased by 1 h, by 2.1 when the surgeon who operated was a senior and by 1.2 when the blood pressure decreased by 10 mmHg. It decreased by 0.6 when the surgery was an arthroscopy.

The risk of respiratory complications was multiplied by 1.7 when the age increased by 10 years, by 1.6 when the weight of the horse increased by 100 kg, 1.5 for each hour of additional anesthesia and 2.6 when the surgeon was a senior. It decreased by 0.7 when the PaO_2_/FiO_2_ ratio increased by one unit.

The risk of systemic complications was multiplied by 1.93 when the ASA score increases by 1 point.

The risk of cardiovascular complications was multiplied by 1.1 each additional year of age of the horse, and by 4.9 when the horse was placed in lateral recumbency; It decreased by 0.5 when the PaO2 / FiO2 ratio increased by one unit.

Odds-ratio of the explanatory variables for the complications are presented in Table 3.

The links between explanatory variables are presented in Appendix 1 (Supplementary Material).

## Discussion

### Mortality Rate

The overall anesthetic mortality rate at the equine veterinary teaching hospital of Lyon was 1.4% overall and 0.96% for non-colic cases. These results are slightly higher than those that were reported by Dugdale et al. (1.1% for all cases and 0.9% for elective cases for the same postoperative period of observation) (3). In 1993, 26 years earlier, Young and Taylor (10) had found an even lower rate for elective cases (0.68%).

Nevertheless, when the observation period was extended to 7 days postoperatively, the published figures of mortality were 1.9% overall anesthesia and 0.9% when colic surgeries were excluded (2). As mentioned in the introduction, it is very difficult to compare the results of studies that differ by many parameters. The need for a multicenter prospective study is still relevant as suggested by Gent and Bettschart-Wolfensberger (12). Horses presented for colic surgery had a very high mortality rate (one third) either because the lesions were inoperable (30%) or due to anesthetic complications (3%). These results lie between those given by Dugdale et al. (1.6%) and those of Mee et al. (4.3%) (3, 13). A possible explanation for this high mortality rate is that colic surgeries are performed on more and more complicated cases. Leisure horses' owners are increasingly willing to operate their horses even if the prognosis is poor.

Many studies showed a significant increase in anesthetic risk for colic surgery (2, 3, 13, 14). In this study, the factors considered as the consequences of the colic syndrome (such as decreased blood pressure, increased ASA score, hypoxemia, hypotension, duration of anesthesia, senior surgeon, emergency, horses not fasted, etc…) also appeared as explanatory factors for mortality, neuromuscular, respiratory or cardiovascular complications. This may explain why colic surgery itself did not appear in the final explanatory models of mortality and morbidity.

### Explanatory Factors for Mortality

Our study showed that increased age was associated to increased anesthetic mortality rate. This is consistent with several studies that showed an increase in risk for horses aged 15, 14, and 12 years, respectively (2, 3, 15). However, the absence of foal in our sample did not allow us to show a higher anesthetic risk in horses <1 month, unlike Johnston et al. (2, 15). Muscle diseases, sarcopenia and osteoarthritic degenerative changes are among the most commonly reported clinical problems in aged horses which appear to be prone to fractures and to difficult recoveries (16). In our study, the age is indeed associated to an increased risk of neuromuscular complications. Age increased with weight, ASA score, and duration of surgery. That is certainly why it did not appear to be a risk factor itself in the final model of anesthetic mortality (which included weight and ASA score) and neuromuscular complications (which included weight and duration of surgery).

The results of this study are in agreement with those of many publications that show a high risk of anesthetic mortality when the ASA score is high in human as well as in animals (3, 9). This could be explained by the fact that the ASA score was linked to many confounding factors such as age, types of surgery, urgency, duration of anesthesia, period of intervention.

Unlike studies by Mee et al. (13) and Dugdale et al. (4), we found that weight is a significant risk factor for anesthetic mortality. Among the confounding factors we found age, colic surgery, PaO_2_/FiO_2_ ratio, blood pressure and duration of anesthesia. Indeed it has been shown that the weight of the horse influences the duration of recovery and increases post-operative complications (17, 18).

The risk of anesthetic mortality was multiplied by 10 during orthopedic surgery. This result is in accordance with Johnston et al. (2). This can be explained by the length of these surgeries that are often long, the urgency, that recoveries are complicated (with a significant risk of fracture), that the ASA score is increased. In addition, the surgeon's seniority was identified as a risk factor in our study, yet a senior surgeon is often involved in orthopedic surgery.

Colic and fracture surgeries are performed urgently in horses having a high ASA score. It is therefore natural to find a higher mortality rate for emergency surgeries as Mee et al. also showed in 1998 (13).

The duration of anesthesia increased the risk of anesthetic mortality and systemic complications. Our data demonstrated that anesthesia lasted longer when the ASA score increased, the latter being a variable in the final model of anesthetic mortality and systemic complications. Anesthesia was also longer when the horse was heavier, during surgery performed by a senior, during orthopedic surgery; all these variables were present in the final model of anesthetic mortality. The literature is divided on this point. Some studies including only emergency surgeries showed an increase in anesthetic risk during long surgery (2, 13). In contrast, Mee's study of non-urgent surgeries showed no significant difference (19).

### Complication Rate

The non-fatal complications rate was of 17%, which is similar to those found in the literature: 16.4% for Kim (14) and 13.7% for Senior et al. (20).

### Nature of Complication

The nature of these complications was predominantly neuromuscular or respiratory.

The majority (92%) of the complications observed before the horse returns to its box occurred during the recovery phase.

We did not observe colic during recovery. Nevertheless, continuing the observation of the records up to 48 h postoperatively, we observed slowing of intestinal transit in 19 horses and other types of colic in 19 other horses, all operated for surgeries other than emergency abdominal surgeries. All resolved medically. Among the horses who underwent colic surgery (*n* = 229), 11 were euthanized as their condition worsened.

Among the other complications described up to 48 h after recovery, we recorded 21 corneal ulcers, 13 peaks of hyperthermia, 7 thrombophlebitis, 3 diarrheas, a case of bronchopneumonia, a case of peritonitis and a case of bilateral nasal discharge and cough. Some horses were euthanized because of the aggravation of their condition: three horses who had undergone fracture surgery for which the reduction was not effective, a case of septic arthritis and a case of dysphagia after guttural pouch surgery. The difference between the nature of complications observed during the period of anesthesia and those observed at the return to the hospitalization box indicates the need to separate these observation periods in future studies.

### Explanatory Factors for Neuromuscular Complications

Our study showed an increase in neuromuscular complications when weight increased. This seems logical since the weight of the body affects the weight worn on the muscles and nerves of the dependent legs during recumbency. The etiology of neuropathy and myopathy syndromes is temporary ischemia due to prolonged pressure on muscle groups (21). Nevertheless, this is disputed by the study of Kim who found that, but did not discuss why, the risk is increased in horses under 500 kg (14).

Maybe for the same reasons, our results showed that lateral recumbency increased the risk of neuromuscular complications in agreement with Johnston et al. (22). Indeed, we observed a series of facial paralysis in horses placed in lateral decubitus (despite the removal of the halter). We also recorded few radial or femoral paresis. Movements of the limb (to massage the shoulder) are now systematically implemented in horses placed in lateral recumbency. The insufflation of the air mattress is also controlled according to the weight of the horse so that it is not too hard.

Furthermore, the risk of neuromuscular complications was multiplied by 1.2 when the blood pressure decreases by 10 mmHg. This reinforces the observations of several authors on the important role of hypotension, in addition to ischemia, in the development of myopathy (21, 23, 24). This was confirmed by the implication of a high hypotension index as a factor favoring neuromuscular complications, as emphasized by the study of Young and Taylor (10). It allowed the evaluation of the intensity and duration of hypotension during anesthesia.

A high ASA score is associated with longer anesthesia and with surgery performed more often by a senior. These two variables are in the final explanatory model of neuromuscular and respiratory complications. This probably explains why the ASA score no longer appeared directly in the explanatory models of these complications. Another possible reason is that ASA 2 and 3 scores were the most represented during orthopedic surgery, the latter being a factor increasing the risk of neuromuscular complications.

The duration of anesthesia multiplied by 1.4 the risk of neuromuscular complications every hour of additional surgery. This can be logically explained, among other things, by the fact that the duration of ischemia is correlated with the risk of myopathy (21).

Hypocalcemia was associated with an increased risk of neuromuscular complications. We could not study this parameter in our final model since the systematic measurement of this variable only concerned colic surgery. However, it would be interesting to know if this parameter would prove to be particularly important. For that, it would also be necessary to extend our duration of observation to be able to observe its effect on the digestive complications. Indeed, it was demonstrated that decreased serum calcium promoted the appearance of ileus (25).

### Explanatory Factors for Respiratory Complications

Our model showed that the risk of respiratory complication increased when the PaO_2_/FiO_2_ ratio decreased and the age increased. In addition, the link between these variables showed that the older the horse, the worse its oxygenation is. Hypoxemia is one of the causes of pulmonary edema (26), which represents 80% of the respiratory complications observed in our study which represented 22 % of total complications, therefore the incidence of pulmonary oedema was 18% of all the complications observed. Despite the aging of the population of anesthetized horses, the management of hypoxemia has improved over the years at our hospital, mainly thanks to the more and more systematic use of salbutamol, the administration of which has been facilitated by the acquisition of a spray system placed in the Y-piece of the circuit. This has certainly led to an overall decrease in respiratory complications by reducing the risk of pulmonary edema.

Our study also showed an increase in respiratory complications associated with heavy weight. Body weight as well as body shape influence arterial oxygenation and alveolar arterial oxygen gradient, increasing the risk of hypoxemia (27).

The duration of anesthesia multiplied by 1.5 the risk of respiratory complications every hour of additional surgery. It is also possible that the duration of anesthesia influenced the severity of atelectasis and therefore hypoxemia and its consequences.

Respiratory complications were strongly associated to colic surgeries. Horses operated for colic often have low plasma protein concentration (28) which promotes the development of pulmonary edema (26). Out of hours surgeries also increased the risk of respiratory complication. This could be explained by the link that exists with colic and emergency surgeries.

Upper respiratory tract obstruction (e.g., laryngeal edema due to low head position during long surgery or nasal edema) is also a potential cause of pulmonary edema.

Nevertheless, in our clinic, the horses are systematically recovered with a naso-pharyngeal or oro-tracheal tube (after colic surgeries) and a vasoconstrictor is administered in the nostrils at the end of the surgery. The endotracheal tubes are removed only when the horse is standing. Upper airway obstruction is therefore an unlikely cause of pulmonary edema in this study.

### Explanatory Factors for Cardiovascular Complications

Lateral recumbency multiplied the risk of cardiovascular complications by 5. The latter was more commonly used in older horses undergoing arthroscopies, orthopedic surgeries or surgeries of the head. Lateral recumbency was also associated to ASA score higher than 3.

We found that age was also part of the explanatory model of cardiovascular complications. Indeed, most of these confounding factors with age are associated with increased anesthetic risk.

### Explanatory Factors for Systemic Complications

The ASA score was the only variable retained in the final model of systemic complications. It was therefore the only explanatory variable of the weakness syndrome (i.e., systemic complications) that we had defined by a prolonged recovery time, the treatment of the horse by more than one injection in the recovery box and prolonged sternal position. Mee et al. also showed an increased risk of systemic complication in horses with high ASA scores (19). Indeed, the American Society of Anesthesiologists (ASA) Physical Status (PS) classification proved to be a very good tools for identifying the animals at a greater risk of anesthesia-related death and complications (9).

### Recovery

Although recovery quality has been associated to factors that also affect mortality (4), Mee et al. found no significant relationship between them (19). To our knowledge our study is the first to show that poor and prolonged recoveries are significantly associated with increased risks of anesthetic mortality and neuromuscular, respiratory and systemic complications. This confirms the reliability of the recovery score used, based on objective descriptors (11). Most complications occurred during recovery unlike Johnston's et al. study that suggested increased fatalities (cardiac arrest) in the early time period of anesthesia (2). This suggests that advances in monitoring and in the safety of anesthesia molecules have shifted the risk period to the recovery box where conditions are more difficult to control.

### Common Explanatory Factors

Among the most common explanatory factors for complications and mortality that we observed in the final models, the surgeon's experience and horse weight appeared 3 times, the ASA score, the duration of anesthesia and age of the horse appeared 2 times. A high PaO_2_/FiO_2_ ratio appeared to decrease the risk of respiratory and cardiovascular complications. Weight, surgeon experience and PaO_2_/FiO_2_ ratio were the least cited factors in the literature (14). It is interesting to note that the most experienced surgeons tended to be responsible for cases with the highest risk and therefore associated to increased mortality and morbidity. Unfortunately, we were not able to prove the same for the anesthetists as the names noted on the anesthesia sheet were often only those of the residents even if a senior had supervised them.

### Bias

One of the biases of our study is the categorization of surgeries. All surgeries in the same category are not necessarily perceived as having the same risk.

The important limitation of this study is obviously the small sample size. To identify risk factors that we could modify to improve outcomes, epidemiological studies have to be prospective and have to include many cases from several centers. Nevertheless, as Senior (1) recommended, the study of cases of a single clinic can also contribute to improving the safety of our equine patients comparing our results to those published by other clinics. It also helps to identify confounding factors. It could at least be as effective as the publication of guidelines (1).

The prospective aspect is very important in order to limit recruitment errors. In this study we could not extend a reliable observation after the horse returned to his box and we could not observe the effect of the recovery method on the risk of complication. The prospective aspect is even more essential to homogenize multicenter studies.

The study of intrinsic and extrinsic factors that determine anesthetic mortality and morbidity is dependent on several definitions, those of mortality and morbidity themselves but also that of their correlation with the anesthetic procedure. The definition of mortality (fatal outcome) is easy, however the definition of morbidity is complex and can give rise to various interpretations.

## Conclusion

This study allowed us to identify the weight, the age of the horse, the surgeon's experience, the ASA score and the duration of anesthesia as risk factors for mortality and anesthetic complication. Unfortunately, among these factors only duration of anesthesia could be improved. These results reinforce those of previous studies and underline the interest of regularly reporting these data. In particular, to recruit them, on a larger scale, in a multicenter prospective study.

## Data Availability Statement

The datasets generated for this study are available on request to the corresponding author.

## Ethics Statement

Ethical review and approval was not required for the animal study because this is a statistical analysis of retrospective data retrieved from the archival database of the clinic where the study was carried out. Written informed consent for participation was not obtained from the owners because this is a statistical analysis of retrospective data retrieved from the archival database of the clinic where the study was carried out.

## Author Contributions

CL, LA, and KP participated in conception of the work, data acquisition and interpretation, and wrote or contributed to the writing of the manuscript and revised the manuscript. LA and KP performed data analysis.

### Conflict of Interest

The authors declare that the research was conducted in the absence of any commercial or financial relationships that could be construed as a potential conflict of interest.
