# Supplementary material for: Risk Factors of Anesthesia-Related Mortality and Morbidity in One Equine Hospital: A Retrospective Study on 1,161 Cases Undergoing Elective or Emergency Surgeries
Source: Front Vet Sci. 2020 Jan 22;6:514. doi: 10.3389/fvets.2019.00514 (PMC6990105; doi:10.3389/fvets.2019.00514)
Supplement: Supplementary file 1 [file Data_Sheet_1.PDF]

	Age	Weight	Breed (number of)	Sex	ASA scores	Number of horses fasted	Number of emergencies	Body position during recumbency	Administration of dobutamine	PaO2/FiO2	Administration of a CRI	Mean Arterial Blood Pressure	Hypotension index	Number of head surgeries	Number of Castrations	Number of colic surgeries	Number of arthroscopies	Number of orthopedic surgeries	Number of surgeries performed by a senior surgeon	Period of the day	Period of the week	Duration of anesthesia	Number of assisted recoveries
Year (from 2012 to 2016)	P *	NS	N* Draft horses		(ASA 3 and 5) N *	P*	NS	NS	NS	P **	P***	N***			N *	NS			N *	NS		NS	N***
Age		P***	P*** poney	P*** gelding and mare	P***	NS	P***	P** lateral	NS	N***	N*** Medetomidine	N**	P*	P***	N***	P***	P***	NS	NS	P*** 20H-8H		P***	P***
Breed		N*** poneys P** Draft horses			NS	N** Race and unknown horses	NS				NS	NS			N** Race horses P**Draft horses	N* Saddle horses and unknown			NS	NS		NS	N*Poneys P* Draft horses
Weight				P*** gelding	NS	P***	NS	NS	P***	N***	N***	NS	N*		NS	P*	P***	N***	NS	P*** 00H-8H		P**	P***
Sex					P*** ASA1-Stallion		N*** Stallions	N*** Lateral Stallions		P * Mare	NS	N* Gelding	NS	N** Stallions	P*** Stallions	N** Stallions	N** Stallions		N***	N*** Stallions			
ASA (From 1 to 5)						N*** ASA 4 and 5	P***	P*** Lateral ASA 1-2-3		N***		P***	P***		P*** ASA 1 and 2	P*** ASA 4 and 5	P*** ASA 2and 3	P*** ASA 2and 3	P***	P***20H-8H	P*** week end	P***	P***
Number of arthroscopy						P***	N***	P***Lateral				NS	NS						P***			P***	NS
Number of colic surgeries						N***	P***	P***Dorsal	NS	N***	P***Lidocaine	P***	P***						P**	P***20H-8H	P*** week end	P***	P***
Number of castration							N***		NS										N***	P***Day	P***week	N***	
Number of orthopedic surgeries						NS	N***	P***Lateral	N***	P***	P*** Medetomidine	P***	N***						P**	P* 8h-20h		P***	P**
Number of head surgeries								P***Lateral		P***										P* 8h-20h			
Number of surgeries performed by a senior surgeon						N*	NS	NS	NS	NS		NS	NS							P* 20h-00h	NS	P***	
Number of emergencies								P**Dorsal	NS		P***Lidocaine	P***	NS							P***20h-00h	P*** week end	P***	P***
Number of horses fasted								P*Lateral		P***		N***	NS							N***20h-8h		N***	NS
Period of the week																						P***	
Period of the day								P*** 00h8h dorsal		N***00h-08h												P*** 8-20H	P***20h-8h00
Body position during recumbency										P*** Lat		NS	N*** Lateral									N*** Lateral	NS
Duration of anaesthesia									P***			P***	P***									N*	P***
Administration of dobutamine													P***										
Mean Arterial Blood pressure													N***										NS

Appendix 1. Statistically significant relationships between the explanatory variables for each category of anesthetic complications (Neuromuscular, Respiratory, Cardiovascular or Systemic) observed in anesthetized horses between January 2012 and December 2016. P: positively correlated, N: negatively correlated. "***": $p<0.001$; "**"$0.001<p<0.01$; "*": $0.01<p<0.05$
